# Genetically Modified Products, Perspectives and Challenges

**DOI:** 10.7759/cureus.7306

**Published:** 2020-03-18

**Authors:** Dimitrios T Karalis, Tilemachos Karalis, Stergios Karalis, Angeliki S Kleisiari

**Affiliations:** 1 Nutrition and Dietetics, University of Thessaly, Volos, GRC; 2 Obstetrics and Gynecology, General Hospital of Trikala, Trikala, GRC; 3 Internal Medicine, General Hospital of Trikala, Trikala, GRC; 4 Nutrition and Dietetics, University of Thessaly, Trikala, GRC

**Keywords:** genetically modified products, environment, genes, diseases, world hunger, substantial equivalence, precautionary clause, safeguard clause, labeling

## Abstract

It is a common ground that humans have always modified the genome of both plants and animals. This intrusive process that has existed for thousands of years, many times through mistakes and failures, was initially carried out through the crossing of organisms with desirable features. This was done with the aim of creating and producing new plants and animals that would benefit humans, that is , they would offer better quality food, more opportunities for people to move and transport products, greater returns to work, resistance to diseases, etc. However, creating genetically modified organisms does not proceed without conflicts. One part of the equation concerns objections made by disputants of genetically modified organisms to the manipulation of life, as opposed to defenders who argue that it is essentially an extension of traditional plant cultivation and animal breeding techniques. There are also conflicts regarding the risks to the environment and human health from using genetically modified organisms. Concerns about the risks to the environment and human health from genetically modified products have been the subject of much debate, which has led to the development of regulatory frameworks for the evaluation of genetically modified crops. However, the absence of a globally accepted framework has the effect of slowing down technological development with negative consequences for areas of the world that could benefit from new technologies. So, while genetically modified crops can provide maximum benefits in food safety and in adapting crops to existing climate change, the absence of reforms, as well as the lack of harmonization of the frameworks and regulations about the genetic modifications results in all those expected benefits of using genetically modified crops being suspended. However, it is obvious that the evolution of genetically modified products is not going to stop. For that reason, research on the impact of genetic modification on medical technologies, agricultural production, commodity prices, land use and on the environment in general, should therefore continue.

## Introduction and background

Biotechnology has developed many procedures that specialize in genetic recombination; the attempt to move genes from one organism to another or to change the genes present in a specific organism results in the expression of new attributes that originally were not there. The above procedures that allow gene alterations of a food or an organism result in Genetically Modified (GM) food or Genetically Modified Organisms (GMO). The concept of gene altering has initiated many debates, with one side criticising the unknown effects and risks on both public health and the environment, and the other supporting the genetic modification's benefits on economy and hunger elimination. This article attempts a literature review on Genetically Modified Products, and specifically the possible risks that they pose, the benefits of their production and use, as well as some basics concepts that have been described and analyzed in current published writings.

## Review

Possible risks of using genetically modified products

Environmental Hazards

There is strong evidence that genetically modified plants appear to interact with their environment [1]. This means that genes introduced into genetically modified plants may be transferred to other plants or even to other organisms in the ecosystem [2-3]. Gene transfer between plants, especially among related plants, results in genetic contamination and is carried out by the transport of pollen [4]. Because natural wild plant varieties are likely to have a competitive disadvantage against genetically modified crops, they may not be able to survive, resulting in the reduction or disappearance of wild varieties [5]. Changing biodiversity worldwide will result in increased resistance of several species of weeds, others to dominate and others to decline or disappear, thus creating a complete and general deregulation in ecosystems [6]. It is a common belief in scientific circles that research needs to be continued to assess the risks and benefits of crops more accurately and adequately.

Risks to Human Health

There may be allergenic effects - especially in people who are predisposed to allergies - or other adverse effects on human health [7]. Experimental studies in animals have shown weight gain, changes in the pancreas and kidneys, toxic effects to the immune system, changes in blood biochemistry among other effects [8,9]. Moreover, the lack of large-scale long-term epidemiological studies that lead to safe conclusions about the allergenic effects of genetically modified plants makes researchers skeptical about the use of genetically modified products. This is because the introduction of a gene that expresses a non-allergenic protein does not mean that it will produce a product without allergenic action. Also, allergies from genetically modified products may be more intense and dangerous, as the allergenic potential of these foods is stronger than that of conventional plants [10,11].

Resistance to Antibiotics

We must note from the outset that the use of antibiotic-resistant genes has stopped in most mutated products. The main problem now lies in the widespread use of antibiotics in feed which, as a natural consequence, end up in the human body through the consumption of dairy products and meat, and thus create resistant germs in the human digestive system [12]. However, more research and studies are needed to determine the differences between transgenic plants from traditional plants and whether genetically modified plants pose additional risks to the consumer public [13,14].

Benefits of using genetically modified products

Hunger Elimination

One of the arguments put forward by advocates of genetically modified products is to eliminate world hunger, a perception that has encountered various reactions [15-16]. A series of extensive and long-term research has shown that the benefits of growing genetically modified crops in the fight against global food shortages and hunger have been significant. The steady increase in the global population has led researchers to focus on the benefits of developing genetically modified products, rather than the potential risks they pose each time [17].

Economic Benefits

A number of studies show the economic benefits of using genetically modified products. Between 1996 and 2011, farmers' income worldwide increased by $92 million from the use of genetically modified crops. Part of the revenue is due to the more efficient treatment of weeds and insects, while another part is due to lower overall production costs. The greatest economic benefits have been achieved in the US, Argentina, China and India, while at the same time, production costs have fallen sharply [18]. At this point, however, there are conflicting reports [19].

Insect Resistance

Bacillus thuringiensis (or BT) is a Gram-positive, soil-dwelling bacterium, commonly used as a biological pesticide. During sporulation, many BT strains produce crystal proteins (proteinaceous inclusions), called δ-endotoxins, that have insecticidal action. This has led to their use as insecticides, and more recently, to genetically modified crops using BT genes, such as BT corn. The main target of these plants is to combat the European Corn Borer insect which is responsible for the destruction of maize crops with a loss of up to one billion dollars a year [20].

Nematode Resistance

Parasitic nematodes are responsible for much of the crop losses. They attack many different plants by destroying the root system. Nematodes, which are essentially a worm species, survive in the soil in very difficult conditions for many years. Chemical control of nematodes is prohibited because there is a high environmental risk. The only natural way to deal with this is through crop rotation (the practice of growing a series of dissimilar or different types of crops in the same area in sequenced seasons), but this is often not possible due to the high financial cost [21]. Thus, the introduction of genes from nematode-resistant plants seems to be the only way to deal with the problem [22]. 

Resistance to Herbicide Round Up

It is common ground that the use of herbicides and pesticides in general causes serious problems for the environment and, consequently, for human health. We know that in areas where wheat is cultivated, that is, where the use of herbicides is increased, the number of child births is clearly decreasing, complications in childbirth occur, and children are born with serious health problems mainly related to mental retardation and autism spectrum [23]. Genetically modified products enable farmers to use a smaller amount of herbicides. Genetically modified soy beans produce an enzyme resistant to the action of the herbicide. The herbicide Round Up destroys the action of a plant enzyme, thereby destroying the plant. Genetically modified plants, however, produce a glyphosate-insensitive form of this enzyme, making it resistant and not affected by the action of the herbicide [24-25]. Researchers are divided on the effects on human health and animals [26].

Cold Resistance

An important advantage of genetically modified plants is the creation of varieties that are resistant to cold temperatures that would normally result in the plant freezing and destroying the plant, thereby losing production. Since the mid-2010s, because of the rapid global change in climate and because plants cannot adapt to rapid temperature changes, scientists have turned to transgenic plants to address the problem [27].

Heat Resistance

In the near future, continuous global warming (as scientists at least claim) will have disastrous consequences for plants, especially in areas where water shortages are already occurring. Creation of modified genes (Sh2 and Bt2) can help plants withstand high temperatures [28-29].

Basic concepts related to genetically modified products

The Notion of Substantial Equivalence

The concept of substantive equivalence has been introduced in the debate on genetically modified products to ensure that these foods are safe [30]. The principle of substantive equivalence holds that if the genetically modified product contains substantially equivalent ingredients present in the conventional product, then no further safety rules are required. In this way the principle of substantial equivalence is a method of evaluating genetically modified products and finding negative factors (such as allergens due to the presence of new proteins) [31,32].

The Precautionary Principle

According to the precautionary principle, any new genetically modified product should not be made available to consumers unless there is first-hand evidence that the product is safe or if there are serious conflicts and conflicting opinions of researchers on the safety of the product in question [33]. Many researchers, however, have argued that the precautionary principle can act as a deterrent to the evolution of science and society, as it may stop or delay any new technology which is capable of solving environmental or economic problems [34]. We should note, however, that criticisms have been raised about the utility and the way the precautionary principle works [35].

The Safeguard Clause

The safeguard clause allows Member States of the European Union to prevent the circulation and sale of genetically modified products which may be harmful to citizens [36].

The Cartagena Protocol

The purpose of this document is to protect the world's biodiversity by instituting stringent rules on the transfer of genetically modified products from one country to another [37].

Labeling of Genetically Modified Products

The appearance of genetically modified products has resulted in the need for labeling of these products [38]. Genetically modified foods should have a special label indicating that they contain genetically modified ingredients. However, as simple as it sounds, the issue of genetically modified products labeling is particularly complex and difficult, as there are important questions about how labeling will be done [39]. For example, it has been argued that products containing either modified protein or foreign DNA should bear a special label. However, there are genetically modified products that do not contain modified protein or foreign DNA, so there is the debate whether these foods, although modified, require special labeling or not. [40].

Ethical Concerns

The key ethical issue regarding the cultivation of genetically modified plants is that the creation of these crops is essentially an interference with the natural flow of life. The ethical dilemma arises as to how to find the middle ground in the use of genetically modified products, given that different countries have different perceptions of the importance of risk, with many countries banning the use of genetically modified products, while companies producing these products focus on profits, and do not take into account the problems that may or may not arise. The problem here focuses on the high degree of uncertainty about the impact of using genetically modified organisms, while the arrangements proposed are usually shaped by financial and political interventions [41]. Consumer attitude is also of particular importance, as consumers are buying and paying their vote of approval at the same time. Consumers are divided into two categories, the consumers who favor the genetically modified organisms and those who oppose them. Consumers' views are influenced by the information they are offered each time, the existing regulations, the confidence they have in the government in regulating the issues that arise, and what they are prepared to pay [42].

Ethics and the Environment

Environmental ethics plays a dominant role in discussions concerning biotechnology and genetic engineering, as many of the arguments presented against genetic engineering have to do with whether it is morally right to genetically modify organisms and the environment, as this may have serious environmental impacts. This shift is evident even in product ads, where companies say environmental protection is a priority for them [43].

Ethics and Animal Rights

Specifically with regard to animals, modern ethical and philosophical considerations hold that animals, like humans, have rights and that these rights should in no way be violated [44]. Animals need to be treated as living organisms and not as commodities or human services. Introducing genes into animals and carrying out experiments can lead to drastic changes in the physiology and behavior of the animal. The results may not be desirable, and in some cases, they may even be disastrous [45].

Patenting Living (Genetically Modified) Organisms

The creation of new organisms inevitably leads to the need to register them and allocate their ownership. But even in the case of registration of a novel product, the 'owner' of the new organism must ensure that the genetic modification does not cause undesirable effects to the environment and humans, as he will be responsible for any problems that may arise [46].

## Conclusions

In recent years there has been enormous technological progress in the creation of genetically modified organisms. There is no doubt that in the future there will be a continuum that will be influenced by both scientific developments and public attitudes towards genetically modified organisms. Creating genetically modified organisms, however, does not proceed without conflicts; there are the disputants of genetically modified organisms who see their production as a manipulation of life, as well as conflicts regarding the risks to the environment and human health. Even though, it is obvious that the evolution of genetically modified crops is not going to stop. Research on the impact of genetically modified crops on agricultural production, commodity prices, land use and the environment in general should therefore continue. Additionally, it is necessary to inform the consumer in order to understand the role of modern technology in crops and agricultural production, and in particular to understand the importance of genetic modifications. In any case, there should be strict and enforceable rules for the use of genetically modified organisms, an assessment of the potential risks of genetically modified crops and clear references to the effects and the results of genetic modifications, both on the environment and on human health.
